# Dendrobium officinale polysaccharide ameliorates polycystic ovary syndrome *via* regulating butyrate dependent gut–brain–ovary axis mechanism

**DOI:** 10.3389/fendo.2022.962775

**Published:** 2022-08-05

**Authors:** Xueping Feng, Decai Wang, Linlin Hu, Haishan Lu, Bo ling, Yanna Huang, Qinyang Jiang

**Affiliations:** ^1^ College of Animal Science & Technology, Guangxi University, Nanning, China; ^2^ College of Basic Medicine, Youjiang Medical University for Nationalities, Baise, China; ^3^ Department of Library, Youjiang Medical University for Nationalities, Baise, China; ^4^ Reproductive Medicine Center, The Affiliated Hospital of Youjiang Medical University for Nationalities, Baise, China; ^5^ Department of Pathology, The Affiliated Hospital of Youjiang Medical University for Nationalities, Baise, China; ^6^ College of Pharmacy, Youjiang Medical University for Nationalities, Baise, China

**Keywords:** dendrobium officinale polysaccharide, polycystic ovary syndrome, ovary, gut microbiota, butyrate

## Abstract

Research has shown that dendrobium officinale polysaccharide (DOP) can promote follicular development and inhibit the apoptosis of ovarian granular cells in PCOS rats. However, DOP cannot be absorbed directly by the stomach and small intestine but is degraded into short-chain fatty acids by gut microbiota in the large intestine and regulates the composition of gut microbiota. How DOP improved ovarian function in PCOS rats through the blood–brain barrier is unclear. In this study, we generated letrozole-induced PCOS rat models and studied the therapeutic effect and mechanism of DOP. 16S rRNA amplicon sequencing analysis, GC-MS short-chain fatty acid detection, and Gene Expression Omnibus database searching were conducted to screen the significantly changed pathways, and a series of experiments, such as enzyme-linked immunosorbent assay, RT-qPCR, Western blot, and immunohistochemistry, were performed. We found that DOP treatment could improve ovarian morphology and endocrine disorders, restore the normal estrus cycle, increase gut microbiota α diversity, and alter β diversity and enrichment of butyrate-producing bacterium in PCOS rats. In addition, compared with PCOS rats, those treated with DOP exhibited higher butyrate and polypeptide YY levels, possibly due to the regulation of G protein-coupled receptor 41 expression. These results indicated that DOP relieved the symptoms of PCOS rats which may be related to the mechanism of butyrate dependent gut–brain–ovary axis protection.

## Introduction

Polycystic ovary syndrome (PCOS) is the most common endocrine and metabolic disorder prevalent in women of reproductive age. It is characterized by ovulatory dysfunction, hyperandrogenism, and polycystic ovarian morphology (1) and is accompanied by various metabolic abnormalities, such as insulin resistance, hyperinsulinemia, and adiposity (2). It has been demonstrated that gut microbiota composition changes and dysbiosis occur in PCOS animal models and women with PCOS, which is closely related to hyperandrogenism (3–5). Gut bacteria-released metabolites have an effective role in weight control by stimulating gut satietogenic hormones, controlling lipid metabolism in adipose tissue, influencing insulin signaling, and improving gut barrier function (6).

Metformin (MET) is an insulin sensitizer, which can improve menstruation, hyperinsulinemia, hyperandrogenemia, and BMI in PCOS (7–10), but long-term use can lead to gastrointestinal distress and even lower pregnancy rates (11, 12). Therefore, traditional Chinese medicine, which has few side effects and a wide range of targets, is becoming an option for the treatment of PCOS.

Dendrobium officinale Kimura et Migo is a traditional and valuable Chinese medicine and is popularly consumed as a functional dietary supplement. Dendrobium officinale polysaccharide (DOP) is the main active component that has various biological functions including antioxidant (13), anti-angiogenesis (14), anti-fatigue (15), and anti-apoptosis (16), as well as improving gut health (17). DOP treatment in type 2 diabetic rats improved the liver metabolism disorder and protected the liver against oxidative stress and inflammatory injury (18). Moreover, DOP could also improve insulin resistance and abnormal lipid metabolism in obese mice (19). Those studies suggest that DOP has great potential in the treatment of diseases related to lipid metabolism abnormalities. Research has shown that DOP can promote follicular development and inhibit apoptosis of ovarian granular cells in PCOS rats (20), but DOP cannot be digested and absorbed by the stomach and small intestine (21), and how DOP acts on the ovary through the blood–brain barrier remains unclear.

Herein, this study was conducted to investigate the effects of DOP on estrus cycle, pathophysiology, endocrine hormone, gut microbial diversity, and metabolites in PCOS rats to clarify the mechanism of DOP improving PCOS.

## Materials and methods

### Materials

Dendrobium officinale polysaccharide (VTY24621, Clara Reagent Grade, 98%) was purchased from Dehang Wuzhou (Beijing, China). Primary antibodies PI3 Kinase p85 (Cat#4257), anti−Phospho-AKT (ser473) (Cat#4060), and anti−Phospho-mTOR (ser2448) (Cat# 5536) were purchased from Cell Signaling Technology (USA). Anti-GPR41 (Cat#AF9057) and anti-GAPDH (Cat#AF7021) were purchased from Affinity Biosciences Technology (Jiangsu, China). SYBR Premix EX Taq™ (RR047A) and SYBR Premix EX Taq™ Kit (RR820A) were purchased from TaKaRa (Dalian, China), and PCR primers were purchased from Gencreate (Wuhan, China). RIPA tissue/cell lysate was purchased from Solarbio Life Sciences (Beijing, China). The ELISA kits [testosterone (E05101m), insulin (E05070r), polypeptide YY (E13432r), and estradiol (E05110r)] were purchased from CUSABIO (Wuhan, China). Metformin hydrochloride (H11021518) was purchased from Beijing Jingfeng Pharmaceutical Group Co., Ltd. (Beijing, China).

### Animals

Eight-week-old female Sprague-Dawley rats (180 ± 20g) were purchased from Changsha Tianqin Biotechnology Co., Ltd. (Hunan, China) [SCXK (Hunan) 2019-0014, No. 430726210100078487] and raised at the Laboratory Animal Center of Youjiang Medical University for Nationalities [SYXK (Guangxi): 2017-0003] (22 ± 2°C, relative humidity 55 ± 5%, 12-h light/dark cycle). All the experimental procedures were approved by the Animal Welfare and Ethics Committee of Youjiang Medical University for Nationalities (YY. No 2020032511).

### Establishment of PCOS model and treatment

After 1 week of acclimatization, vaginal smears were taken for 5 consecutive days, and 30 rats with normal estrus cycles were selected for subsequent experiments. Then the rats were randomly divided into two groups: the normal saline group (NS group) and the model group. The model group was orally administered with letrozole [1 mg/kg/day, dissolved in 0.5% carboxymethyl cellulose (CMC)], and the normal saline group was orally administered with 0.5% CMC (1 ml/kg/day) for 28 days. Three rats were randomly selected from each group for model validation, and the successful PCOS model in rats was ensured by testing serum testosterone levels and ovarian histopathological observations. Then the model group was randomly divided into dendrobium officinale polysaccharide group (DOP group), metformin group (MET group), and polycystic ovary syndrome group (PCOS group). For the PCOS group, the rats were orally administered with normal saline (1 ml/kg/day). For the DOP group, the rats were orally administered with DOP (200 mg/kg/day). For the MET group, the rats were orally administered with MET (300 mg/kg/day) and for the NS group, and the rats were orally administered with normal saline. During treatment, vaginal smears were performed for 10 consecutive days. After 28 days of treatment, all rats were sacrificed, then fasting blood glucose (FBG), serum, ovaries, colons, and colon feces were collected for further analysis. During the process and treatment, the rats were weighed weekly.

### Serum collection and hormone level determination

After 28 days of treatment, all rats were anesthetized with isoflurane and blood was taken from the abdominal aorta, left at 4°C overnight, then the serum was isolated by centrifuging at 4,000 rpm for 10 min then stored at -80°C. FSH and LH levels were measured by the Beijing North Institute of Biotechnology Co., Ltd. (Beijing, China). The serum concentrations of hormones (testosterone, fasting insulin, polypeptide YY, and estradiol) were determined using ELISA kits.

### Fecal sample collection and determination

Feces were collected from the colon of rats and frozen in liquid nitrogen for 5 min then stored at -80°C. The 16S rRNA amplicon sequencing and GC-MS short-chain fatty acids were detected respectively by OE Biotech Inc. (Shanghai, China) and Luming Biotech Inc. (Shanghai, China).

### Ovary morphology analysis

The ovarian tissue samples were fixed in 4% paraformaldehyde solution at 4°C over 24 h, then embedded in paraffin and cut into 3~4-μm thickness for hematoxylin–eosin staining. Analysis was performed using a light microscope (Olympus, Japan).

### Quantitative real-time PCR analysis

Total RNA was prepared from frozen rat ovarian tissues using the TRIzol method, and cDNA was synthesized using a PrimeScript RT Reagent Kit with gDNA Eraser. Quantitative real-time PCR was performed according to the SYBR Premix EX Taq™ kit instruction and run on the LightCycler 96 system (Roche, USA). The housekeeping gene β-actin was used for normalization, and the relative expressions of each gene were calculated by the 2^−ΔΔCT^ method. Gene primers are listed as follows: β-actin—forward 5′-CGATGGGAAGTGCTGGATAG-3′ and reverse 5′-CGGTTAGAGTAGGTGACGTTG-3′; PI3K—forward 5′-AATACACCTGGTGCTCGACAC-3′ and reverse 5′-CCTCTGATCTTGACCCTGAAC-3′; AKT—forward 5′-CACAGGTCGCTACATGCCA-3′ and reverse 5′-GTAAGGAAGGGATGCCTAGAG-3′; mTOR—forward 5′-TGCCAACTACCTTCGGAACC-3′ and reverse 5′-GCTCGCTTCACTTCAAACTCC-3′; CYP17A1—forward 5′-ATCCGAGAAGTGCTGCGTATC-3′ and reverse 5′-GGCATGAACTGATCTGGCTG-3′.

### Western blot analysis

Appropriate amount of ovarian tissue was added into RIPA tissue/cell lysate solution to extract total protein and determine the concentration. Then, 4× protein loading buffer was added and boiled to denature. Thirty-microgram protein samples were separated with 8% SDS-PAGE gel at 120 V for 1 h. The isolated proteins were transferred onto PVDF membranes and incubated at 250 mA for 1 to 2.5 h. After incubation of the PVDF membranes with the blocking solution, the protein strips were mixed with primary antibody (diluted concentration, 1:1,000) and incubated overnight at 4°C. The blots were then washed with TBST buffer and incubated with goat anti-rabbit antibodies for 1 h at room temperature. The chemiluminescent assay kit and the protein expression levels were normalized to GAPDH. ImageJ software was used to analyze the results.

### Immunohistochemical analysis

The procedure of immunohistochemical staining was carried out according to the instructions. To put it simply, paraffin sections of colon tissue are first dewaxed and hydrated, followed by an antigenic repair, followed by dripping of an endogenous peroxidase blocker, incubated at room temperature for 10 min, washed with PBS for 3 min × 3 times, and finally dropped with primary antibody at 4°C and incubated overnight. On the second day, they are washed with PBS buffer for 3 min × 3 times, incubated with reaction enhancement solution at 37°C for 20 min, and washed with PBS buffer for 3 min × 3 times. Goat anti-rabbit lg G polymer was dropped, followed by DAB display, tap water washing, hematoxylin staining solution incubation for 20 s, alcohol dehydration, xylene transparency, and neutral gum sealing. The staining results were observed under a light microscope.

### Statistical analysis

Data are presented as the means ± standard deviation (SD). Data analysis and mapping were performed separately using SPSS 20.0 and GraphPad Prism 8.0 software. Integrated optical density was measured using Image Pro Plus 6.0 software. Differences among multiple groups were compared using one-way analysis of variance followed by LSD (L)-Dunnett’s T3(3) analysis; *p* < 0.05 was considered to indicate a statistically significant difference.

## Results

### DOP treatment reduced the body weight in rats

**Figure 1:**
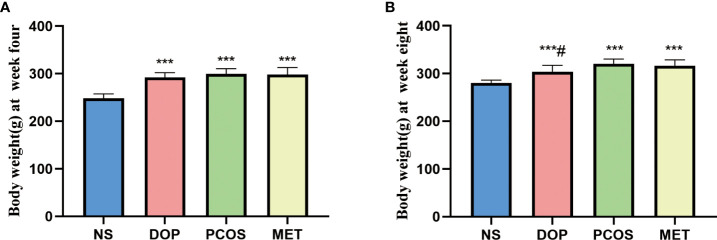
Effects of DOP treatment on body weight in rats, n = 6. Note: compared with the NS group, ***p < 0.001; compared with the PCOS group #*p* < 0.05.

Before the experiment starts, there was no significant difference in body weight between the NS group and model group (**Supplement 1**). After rats were orally administered with letrozole for 4 weeks, the body weight of rats was significantly higher than in the NS group (**Figure 1A**). After treatment for 4 weeks, the body weight of rats in the DOP group was significantly lower than in the PCOS group (**Figure 1B**), indicating that DOP has a certain effect on body weight loss.

### DOP treatment could restore the estrus cycle in rats

The estrous cycle in rat averages 4~5 days and is generally divided into four stages: proestrus, estrus, metestrus, and diestrus. The stages of estrus are distinguished by identifying different cell types (22). Proestrus: nucleated epithelial cells are the majority, oval in shape, and occasionally there are a few white cells and irregular keratinized epithelial cells. Estrus: most of them are irregular-shaped keratinized epithelial cells, which gather together in piles and are shaped like leaves. Metestrus: keratinized epithelial cells, nucleated epithelial cells, and leukocytes are all visible in similar proportions. Diestrus: many neutrophils and a few nucleated epithelial cells are present. During the treatment, vaginal smears were examined in rats for 10 consecutive days (approximately two estrus cycles) at 8 to 9 a.m. from day 19 to assess the effect of treatment (**Figure 2-1**), and it was found that the NS rats still maintained regular estrous cycles (**Figure 2-2A**), while the PCOS rats remained in the diestrus (**Figure 2-2B**), but rats treated with DOP/MET had improved estrus cycles (**Figures 2-2C-D**). A normal estrus cycle should have four consecutive estrus stages; thus, we counted the estrus stages for each rat. A complete estrus cycle observed is defined as estrus regular and conversely as irregular. The results showed that rats in the NS group all had regular estrous cycles but not in the PCOS group. After DOP/MET treatment which can reverse to irregular in most of the rats, the recovery rate of estrus cycles was up to 66.7% and 83.3% (**Figure 2-2E**).

**Figure 2-1:**
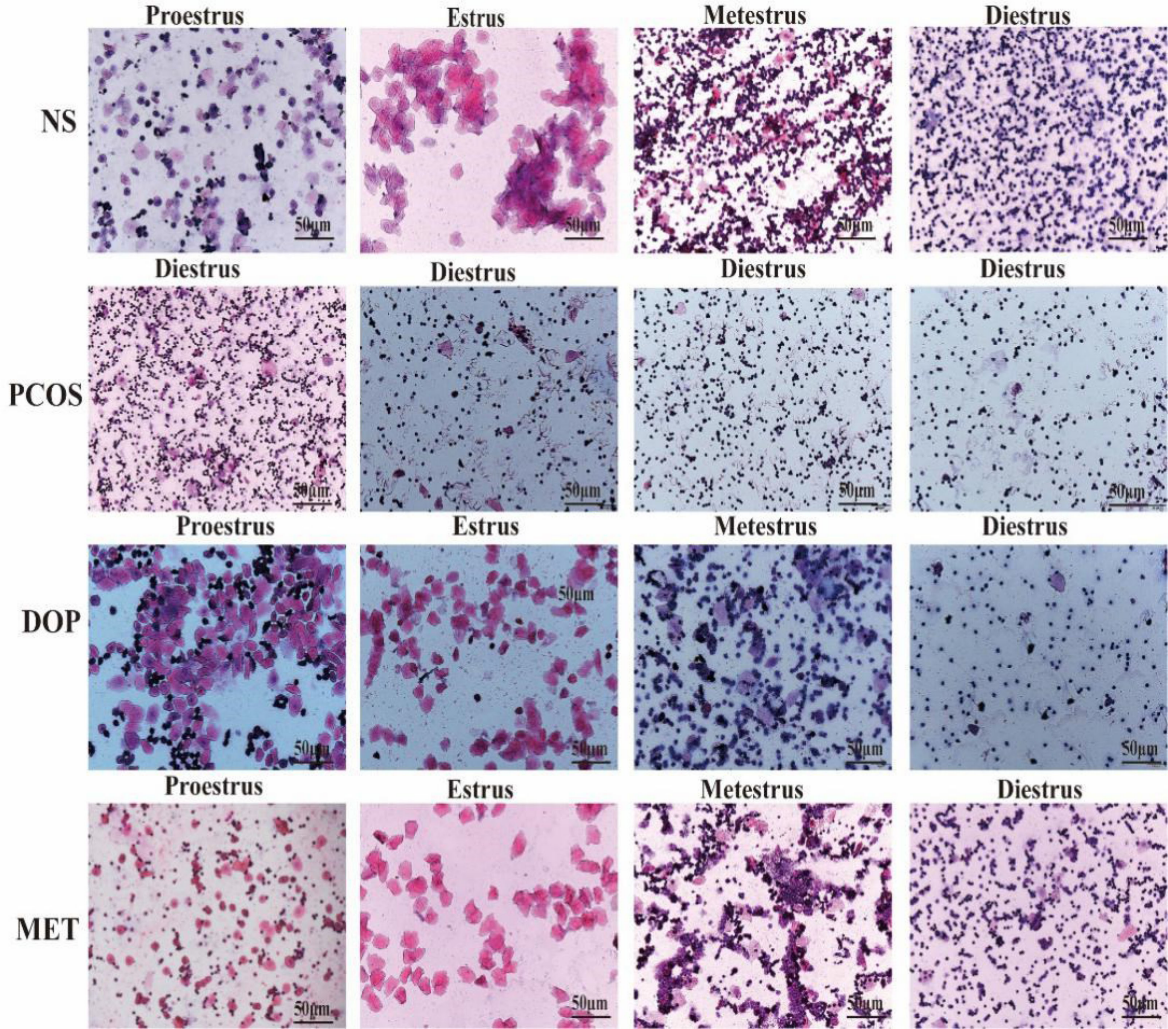
Vaginal smear identification of estrus stage in rats **(H&E)**. Note: scale bar = 50 μm.

**Figure 2-2:**
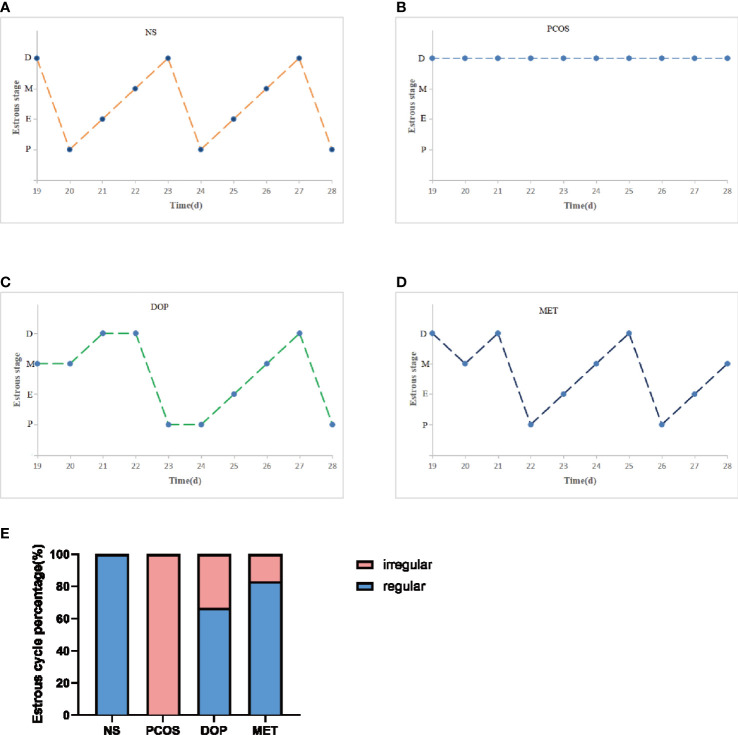
Effects of DOP treatment on estrus cycle in rats, n = 6. **(A–D)** Rats were examined at the estrus stage for 10 consecutive days. D: diestrus, M: metestrus, E: estrus, P: proestrus. **(E)** The proportions of regular and irregular estrus cycles in each group of rats.

### DOP treatment improved endocrine disorders but without affecting fasting glucose in rats

**Figure 3:**
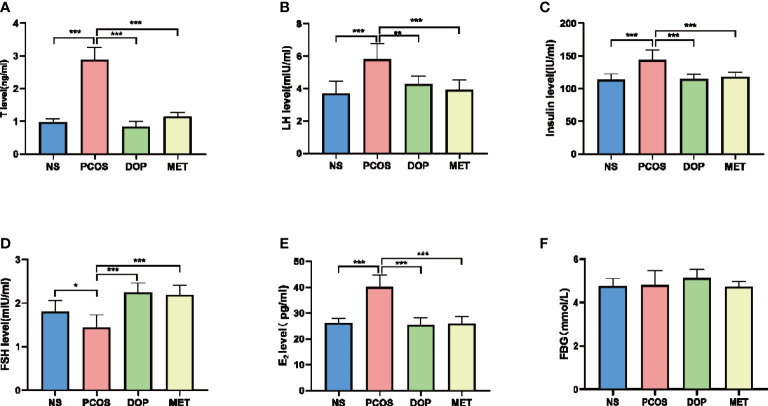
Effects of DOP treatment on sex hormone levels and fasting blood-glucose (FBG) in rats, n = 5–6. **(A)** T, **(B)** LH, **(C)** insulin, **(D)** FSH, **(E)** E_2_, and **(F)** FBG. Note: compared with the PCOS group, *p < 0.05, **p < 0.01, ***p < 0.001.

To evaluate the therapeutic effects of DOP, we collected serum for steroid hormone level detection. The testosterone (T), luteinizing hormone (LH), insulin, and estradiol (E_2_) levels in the PCOS group were significantly higher than in the NS group (**Figure 3A–C**, **3E**), and DOP/MET treatment was significantly reduced, while the serum FSH levels in the PCOS group were significantly lower than those in the NS group (**Figure 3D**), and DOP/MET treatment significantly increased the serum FSH level when compared to the PCOS group. There was no significant difference in fasting blood glucose among all groups (**Figure 3F**).

### DOP treatment can improve the morphology of polycystic ovary in rats

**Figure 4:**
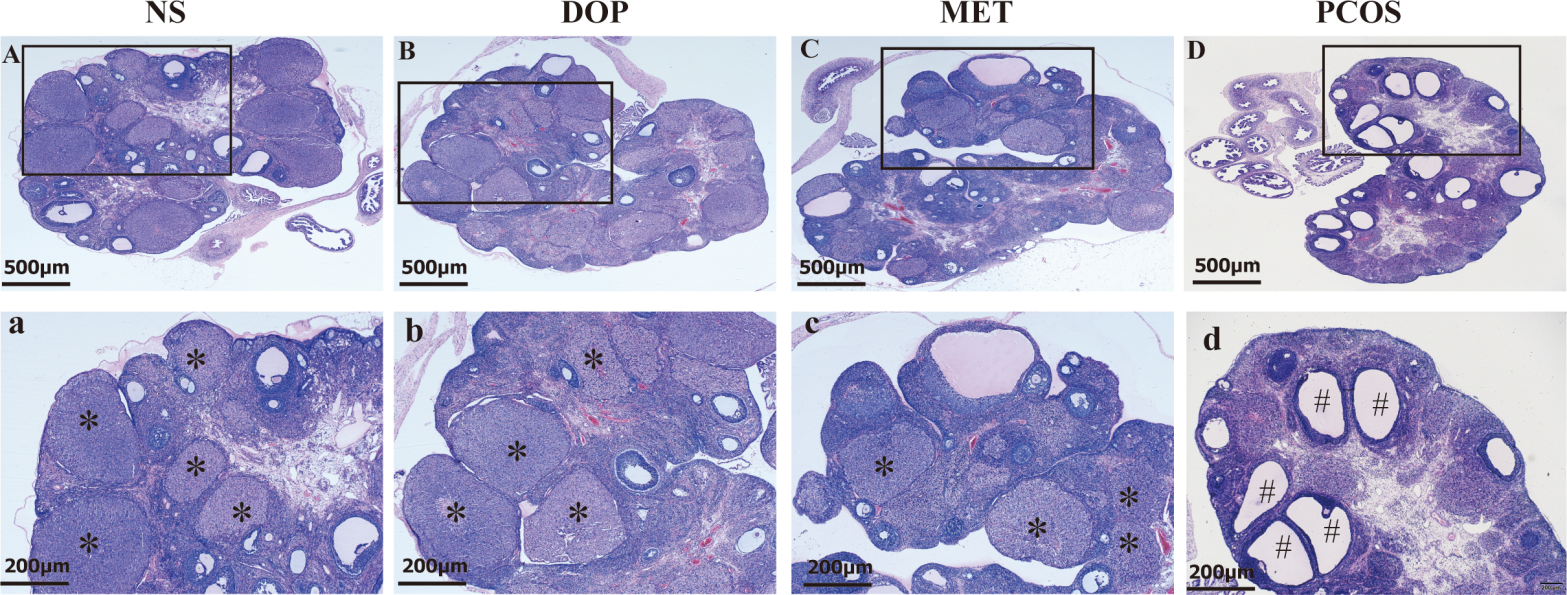
Effect of DOP on the morphology of ovarian pathological tissue in rats. Note: * for corpus luteum, # for cystic follicle, scale bar: **(A–D)** = 500 μm, a-d = 200 μm.

As shown in **Figure 4**, the ovarian morphology in the NS group was normal (**Figures 4A–a**). In contrast, the ovaries of letrozole-treated rats showed typical PCOS characteristics, including increased cystic follicles, fewer corpora lutea, and thinning of granulosa cell layers (**Figures 4D–d**). After DOP/MET treatment, we found that polycystic ovary morphology was markedly attenuated and showed more corpora lutea (**Figures 4B–b, C–c**).

### DOP treatment regulated the PI3K–AKT–mTOR pathway in rats

**Figure 5:**
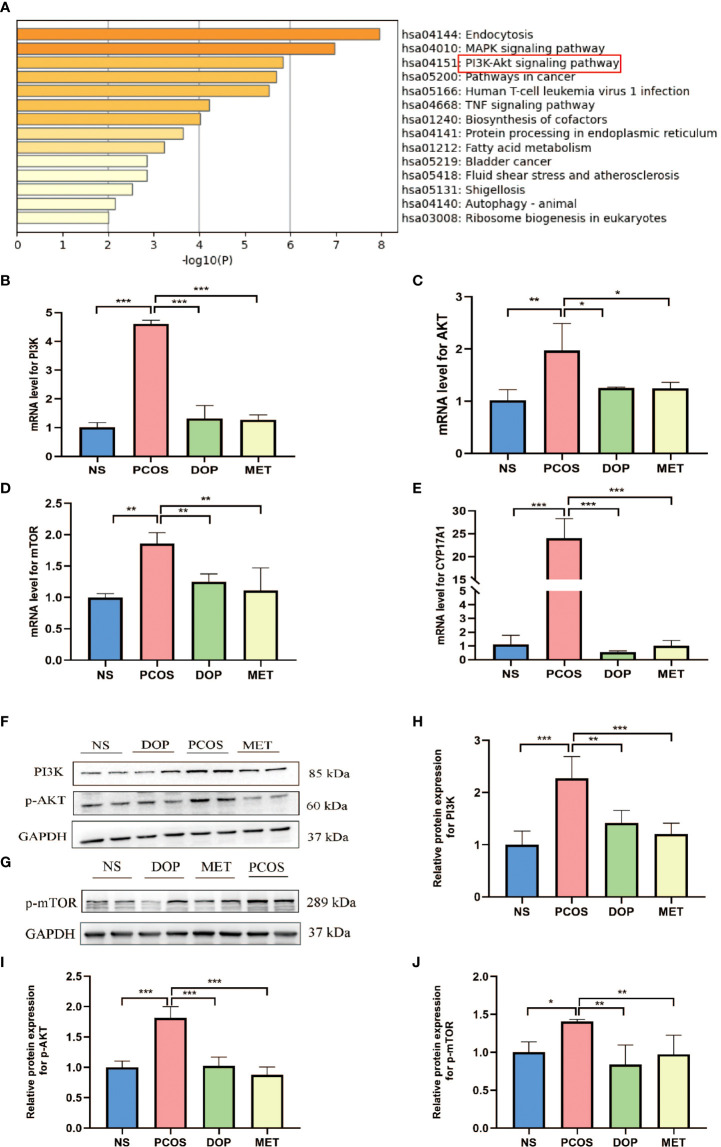
Effects of DOP treatment on the PI3K–Akt–mTOR signaling pathway in rats by qPCR and Western blot. **(A)** Top 14 clusters from Metascape pathway enrichment analysis differently expressed associated genes. Significance is indicated by the −log10 (P) value. **(B–E)** Ovarian gene PI3K, Akt, mTOR, and CYP17A1 expression levels, n = 3. **(F–J)** Expressions of PI3K, p-Akt, and p-mTOR protein are expressed as the fold change in the optical density of a target protein, and GAPDH expression served as the control, n = 2. Note: compared with the PCOS group, *p < 0.05, **p < 0.01, ***p < 0.001.

In order to study the molecular mechanism of DOP treatment to improve the phenotype of PCOS rats, we extracted data set GSE98595 from the GEO database and predicted the differently expressed genes. Metascape was used for gene enrichment analysis (**Supplement 2**). As shown in **Figure 5A**, the first three gene sets with the most significant enrichment were hsa:04144, hsa:04010, and hsa:04151. It is noteworthy that the PI3K-Akt signaling pathway regulates the activation of primordial follicles (23). The results showed that PI3K, AKT, mTOR, and CYP17A1mRNA expression in the PCOS group was significantly higher than that in the NS group (**Figures 5B–E**). After DOP/MET treatment, PI3K, AKT, mTOR, and CYP17A1mRNA expression was significantly lower than in the PCOS group. Western blot results further confirmed that compared with the PCOS group, the expression of PI3K, p-AKT, and p-mTOR proteins in the DOP group was significantly reduced (**Figures 5F–J**).

### The diversity of the gut microbiota was altered by DOP treatment in rats

**Figure 6:**
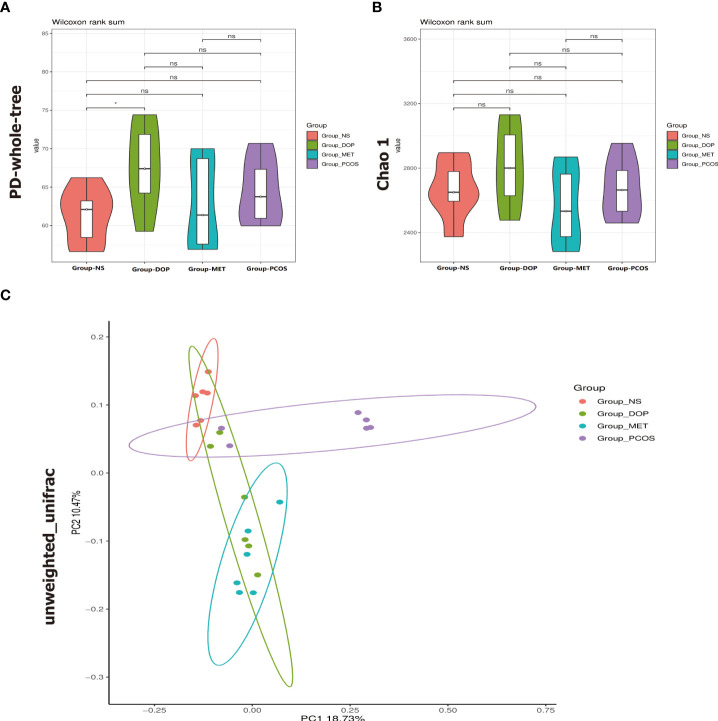
Effects of DOP treatment on gut microbial diversity in rats, n = 6. **(A)** Phylogenic diversity whole tree index (PD_whole_tree index). **(B)** Chao1 index. **(C)** Principal coordinate analysis (PCoA) using the unweighted_unifrac_distance. Note: compared with the NS group, ns *p* > 0.05, * *p* < 0.05.

As mentioned above, our study has proved that DOP can improve endocrine disorders and follicle development in PCOS rats, but DOP cannot be directly absorbed by the stomach and small intestine, which finally degraded into SCFAs by gut microbiota in the large intestine, and the composition of gut microbiota was modulated (21). How DOP affects endocrine and ovarian function in PCOS rats *via* the blood–brain barrier is unclear. Due to these reasons, the V3–V4 regions of the 16S rRNA gene were sequenced in 24 fecal samples to analyze. PD_whole_tree index and Chao1 index were used to compare the gut microbiota α diversity among the four groups. It was found that the gut microbiota α diversity in the DOP group was significantly increased compared with the NS group (**Figure 6A**), but based on Chao1 index analysis, there was no significant difference in α diversity (**Figure 6B**). The overall distribution of gut microbes was assessed based on β diversity, which was calculated using PCoA (**Figure 6C** and **Supplement 3**). A one-way analysis of similarities (ANOSIM) test was used to evaluate the similarities between groups. The results showed that there were significant differences in gut microbiota composition among all groups (ANOSIM *R* = 0.586, *p* = 0.001).

### DOP treatment ameliorated the gut dysbiosis in rats

**Figure 7:**
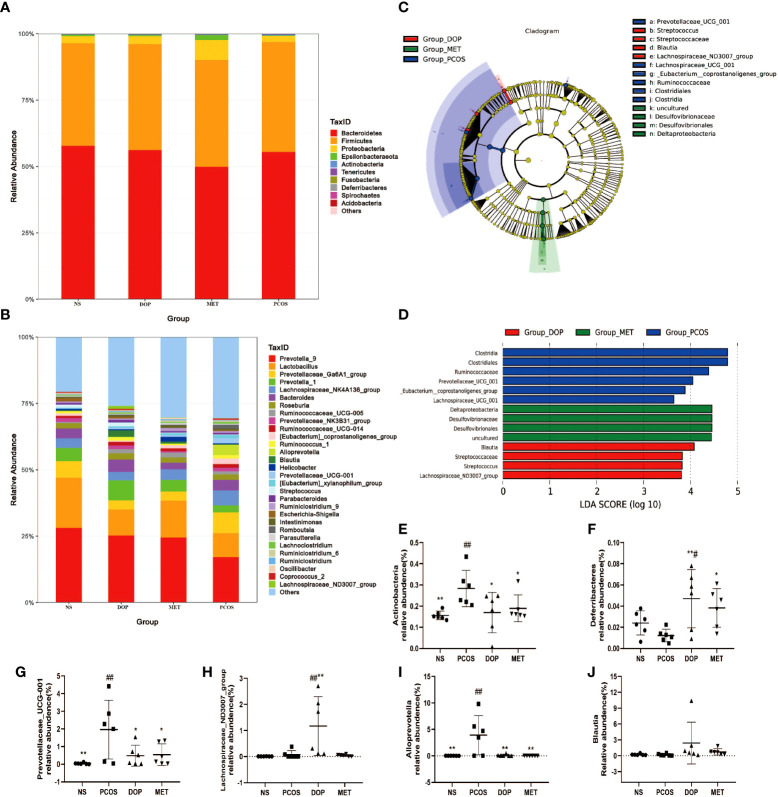
Effect of DOP treatment on gut microbial composition, n = 6. **(A)** Microbial distribution at the phylum level. **(B)** Microbial distribution of the top 30-genus level. **(C)** Cladograms representing the linear discriminant analysis effect size (LEfSe) results. **(D)** Linear discriminant analysis (LDA) results between different experimental groups. LDA scores above 3.00 and *p* < 0.05 are shown. **(E-J)** Relative abundance of *Actinobacteria*, *Deferribacteres*, *Prevotellaceae_UCG_001*, *Lachospiraceae_ND3007_group*, *Alloprevotella*, and *Blautia* between all groups. Note: compared with the PCOS group: * *p* < 0.05, ** *p* < 0.01; compared with the NS group: # *p* < 0.05, ## *p* < 0.01.

Microbial community structure and its metabolites affect animal health. At the phylum level, there were no taxonomic differences observed between all groups and more than 97% of gut bacteria are *Firmicutes*, *Bacteroidetes*, and *Proteobacteria* (**Figure 7A**). At the genera level, the 30 most abundant gut bacteria are shown in **Figure 7B**. To further study the different microbial compositions in the feces of rats in different groups, we constructed a cladogram to show its evolutionary characteristics (**Figure 7C**), and LefSe analysis was performed to distinguish microbial biomarkers (**Figure 7D**). Compared with the NS group, the PCOS group showed an increase in the prevalence of *Actinobacteria*, *Prevotellaceae_UCG_001*, and *Alloprevotella* (**Figures 7E, G, I**). Notably, bacteria from the *Blautia* and *Lachospiraceae_ND3007_group* genera were most significantly enriched in the DOP group (**Figures 7H, J**). Compared with the PCOS group, *Deferribacteres* were more abundant in the MET group (**Figure 7F**). The results showed that there were more harmful bacteria enriched in the intestinal of PCOS rats, such as *Prevotellaceae_UCG_001*, *Ruminococcus*, and *Clostridiales*, and DOP/MET treatment increased the abundance of short-chain fatty acid bacteria, such as *Blautia*, *Lachospiraceae_ND3007_group*, and *Deferribacteres*.

### DOP treatment elevated butyrate levels in feces in rats

**Figure 8:**
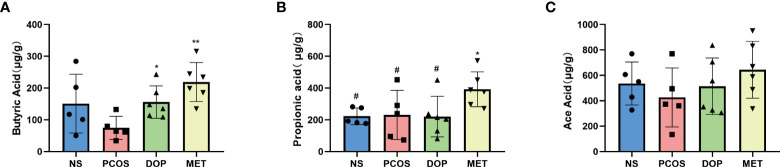
Effect of DOP treatment on fecal short-chain fatty acid (SCFA) levels, n = 5–6. Note: compared with the PCOS group *p < 0.05, **p < 0.01; compared with the MET group #*p* < 0.05.

As the abundances of SCFA producers (such as *Blautia*, *Lachospiraceae_ND3007_group*, and *Desulfovibrionaceae*) in the gut microbiota were enriched, the fecal levels of SCFAs were monitored. Compared with the PCOS group, the levels of fecal butyric acid in the DOP and MET groups were significantly increased (**Figure 8A**), while propionic acid levels were significantly increased only in the MET group (**Figure 8B**). There was no significant difference in the fecal levels of acetic acid among all groups (**Figure 8C**).

### DOP treatment attenuated PCOS through a butyrate dependent gut–brain mechanism

**Figure 9:**
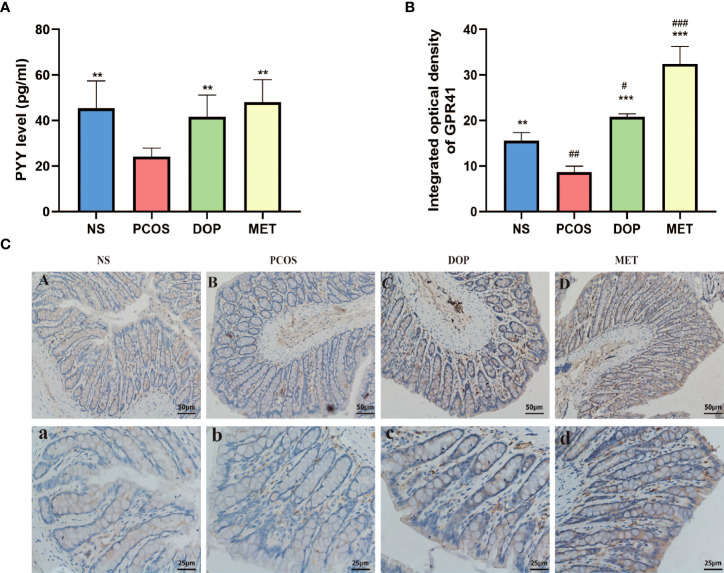
Effects of DOP treatment on gut–brain mediators. **(A)** Serum levels of PYY, n = 5. **(B-C)** Colonic GPR41 expression, location, and integrated optical density, n = 3, scale bar = 50 and 25 μm. Note: compared with the PCOS group, **p < 0.01, ***p < 0.001; compared with the NS group, #*p* < 0.05, ##*p* < 0.01, ###*p* < 0.001.

As previously mentioned, the levels of butyric acid in rat feces were higher after DOP/MET treatment. To determine whether butyric acid mitigates PCOS phenotypes through the gut–brain axis, we assessed the levels of brain–gut regulators peptide tyrosine-tyrosine (PYY) and G protein-coupled receptor 41 (GPR41) in rats. Compared with the PCOS group, the levels of serum PYY in the NS, DOP, and MET groups were all significantly increased (**Figure 9A**); the colonic GPR41 expression and integrated optical density were also significantly increased in the NS, DOP, and MET groups (**Figures 9B, C**). The results showed that the improvement mechanism of DOP on PCOS rats was closely related to the regulation of PYY and GPR41.

## Discussion

Polycystic ovary syndrome is one of the leading causes of anovulatory infertility in women. It has been suggested that the main causes of PCOS are genetic and environmental interactions. For example, an unhealthy lifestyle, diet, or any infectious agent can increase the risk of developing PCOS.

Letrozole is an effective aromatase inhibitor that can block the transformation of androgen to estrogen *in vivo* and is widely used in the construction of PCOS rat models, which can produce endocrine characteristics and ovarian morphological changes similar to clinical PCOS, including hyperandrogenemia, ovulation disorder, and obesity (24). In our study, the body weight and testosterone levels were significantly higher in PCOS rats; after DOP treatment, the body weight and testosterone significantly decreased, which was consistent with previous findings (20, 25). Studies have shown that androgen has strong assimilative activity, promoting food intake, energy absorption, and fat storage. Body weight loss in DOP rats may be closely related to decreased testosterone levels.

Hyperandrogenemia is one of the main features of polycystic ovary syndrome. High androgen and E_2_ levels in serum are not conducive to the growth of dominant follicles and inhibit ovulation (26). In our study, more cystic follicles were observed in the ovaries of rats in the PCOS group, and the normal estrus cycle was lost. The compensatory hyperinsulinemia is thought to be a major driver of hyperandrogenemia in polycystic ovary syndrome which amplifies luteinizing hormone-mediated androgen synthesis. Therefore, metformin is often used to improve insulin sensitivity in patients with polycystic ovary syndrome. Our results showed that MET treatment decreased serum insulin, testosterone, E_2_, and LH levels and promoted the secretion of FSH in PCOS rats, while DOP treatment showed similar effects to MET, indicating that DOP has the effect of improving endocrine disorder in PCOS rats.

The PI3K/AKT/mTOR pathway is closely related to follicular development (23). In PCOS mouse models, it was observed that excessive androgen upregulation of mTORC1 resulted in dominant follicular selection disorder and follicular dysplasia (27), while rapamycin blocked the central mTOR signaling, leading to inhibition of the gonadal hormone axis and significant reduction in LH and estradiol levels in puberty rats (28). Previous studies have shown that metformin can promote the expression of glucose transporter 4, inhibit the expression of the androgen receptor, block the insulin receptor/PI3K/AKT/mTOR signaling pathway, and improve the abnormal metabolism in PCOS (29). 17-A hydroxylase (CYP17A1) plays a key role in steroid synthesis by converting the progesterone to androgen and thereby increasing the level of androgen. Clinical studies suggest that enhanced CYP17A1 enzyme activity and expression may account for hyperandrogenism in PCOS (30). LH stimulates CYP17A1 mRNA expression and androgen production in theca cells *via* activation of the PI3K/Akt pathway (31). Therefore, it was observed in this study that the high secretion of LH may be the cause of the high expression of CYP17A1 mRNA and the elevated testosterone level. In the present study, we observed increased CYP17A1 gene expression and activation of the PI3K/AKT/mTOR signaling pathway and that multiple cystic follicles appear in the PCOS group; after DOP and MET treatment, CYP17A1 and PI3K/AKT/mTOR pathway were downregulated, and the morphology of the polycystic ovary was improved.

PCOS is a complex endocrine and metabolic disease, and there has been increased interest in the interplay with gut dysbiosis. Although our study has proved that DOP can improve endocrine disorders and follicle development in PCOS rats, DOP cannot be digested by enzymes encoded by the human genome. How DOP affects endocrine and ovarian function in PCOS rats *via* the blood–brain barrier is worthy of further study. As a dietary fiber, DOP can be degraded into SCFAs by gut microbiota in the large intestine and modulate the composition of gut microbiota (21). A high-fiber diet can remodel the gut microbiota and relieve chronic metabolic inflammation, reproductive function, and brain–gut polypeptide secretion in PCOS patients (32). Thus, we collected rat feces for 16S rRNA and SCFAS detection, trying to find the detailed mechanism of DOP improving PCOS.

In **Figure 6**, compared with the NS group, there is no significant difference in the PCOS group while DOP treatment can significantly increase the α diversity. PCoA analysis found that β diversity of the gut microbiota has a significant difference between fecal levels from the NS group and PCOS group which could be partially restored by DOP/MET treatment. These data indicated that DOP/MET treatment could reshape the gut microbiota community structure of PCOS rats. Studies have reported that women with PCOS had decreased α diversity and altered β diversity and gut microbiota composition (33, 34). Dysbiosis of the gut microbiota in the host can activate the immune system and interfere with the function of insulin receptors, causing hyperinsulinemia, increasing the production of androgen in the ovary, and preventing the development of normal follicles (35).

LEfSe analysis found that the abundance of beneficial bacteria such as *Blautia* and *Lachospiraceae_ND3007_group* was higher in the DOP group. *Blautia*, a bacterial group belonging to the family *Lachnospiraceae* of phylum Firmicutes, could ferment carbohydrates and produce acetate and butyrate which has been reported to be negatively correlated with obesity and T2D (36, 37). Decaffeinated green and black tea polyphenols reduced body weight in diet-induced obese mice which was strongly associated with *Blautia* enrichment in the gut (38). *Lachospiraceae_ND3007_group* has been reported to be negatively correlated with testosterone (39). These could reasonably explain why body weight and testosterone levels in PCOS rats decreased and butyric acid levels increased after DOP treatment. In the PCOS group, the abundance of *Prevotellaceae UCG-001*, *Ruminococcus*, and *Clostridiales* was higher. According to reports, *Prevotellaceae UCG-001* is thought to be associated with impaired glucose tolerance (40). *Prevotella copri* is considered to be the main species driving the link between branched-chain amino acid biosynthesis and insulin resistance, exacerbating glucose tolerance and increasing circulating levels of branched-chain amino acids (41). *Ruminococcus* is a kind of Gram-positive anaerobic bacteria, which can secrete β-glucuronidase, destroy colonic mucosa, and participate in the invasion and metastasis of tumors (42). Moreover, *Clostridiales* which are significantly enriched in T2D women (43) can metabolize peptones and amino acids to produce lactic acid as the major product (44). In our study, the rats in the PCOS group were observed to exhibit higher T, insulin, and lower butyric acid levels. The intestine is an important organ for metformin’s pharmacological effects, promoting glucose uptake and lactic acid production by influencing the composition of gut microbiota (45). Therefore, the possibility exists that the metabolic benefits linked to metformin treatment may in part depend upon its action in the gut. In the intestines, metformin does not only improve the glucose uptake in T2D individuals, reshape the human microbiota, promote the growth of beneficial bacterial species, and counteract the expansion of detrimental bacterial species but also promotes the short-chain fatty acid (SCFA) production, protects the intestinal barrier, and regulates the secretion of gut peptides (46, 47). In this study, *Desulfovibrionaceae* and *Desulfovibrionales* in the MET group are the dominant genera. Interestingly, *Desulfovibrionaceae* and *Desulfovibrionales* are sulfate-reducing bacteria that can break down lactic acid produced by the microbial community into propionic and butyric acids, which are known to contain mechanisms for combating oxidative stress (48). This may explain why higher levels of propionic and butyric acid were detected in the MET group.

In this study, we observed that levels of butyrate were lower in the PCOS group while higher in the DOP group and MET group. Interestingly, lower butyrate levels were also detected in PCOS patients (49), suggesting a correlation between altered butyrate levels and PCOS (50). In addition, reduced butyric acid levels in diet-induced obesity are an important cause of reduced insulin resistance (51). Butyrate has been shown to act on G protein-coupled receptors (GPR41 and GPR43), resulting in GLP-1 and PYY secretion and thereby regulating sex hormone levels to ameliorate PCOS (52, 53). PYY is secreted by the entero-endocrine L-cells of the distal ileum and colon which may affect sex hormone levels by crossing the blood–brain barrier and binding to neuropeptide Y receptors (54). Studies have reported that PCOS patients have lower PYY, which is negatively correlated with LH and insulin (33, 49). In this study, the levels of serum PYY were lower, but LH and insulin were higher in PCOS rats. Notably, DOP/MET treatment reversed these hormone levels.

This evidence suggests that the ameliorative effect of DOP on PCOS rats is mainly due to promoting the release of butyrate by gut microbiota, which affects the secretion of sex hormones through the butyrate-GPR41-PYY mechanism, rather than DOP itself.

## Conclusions

**Figure 10:**
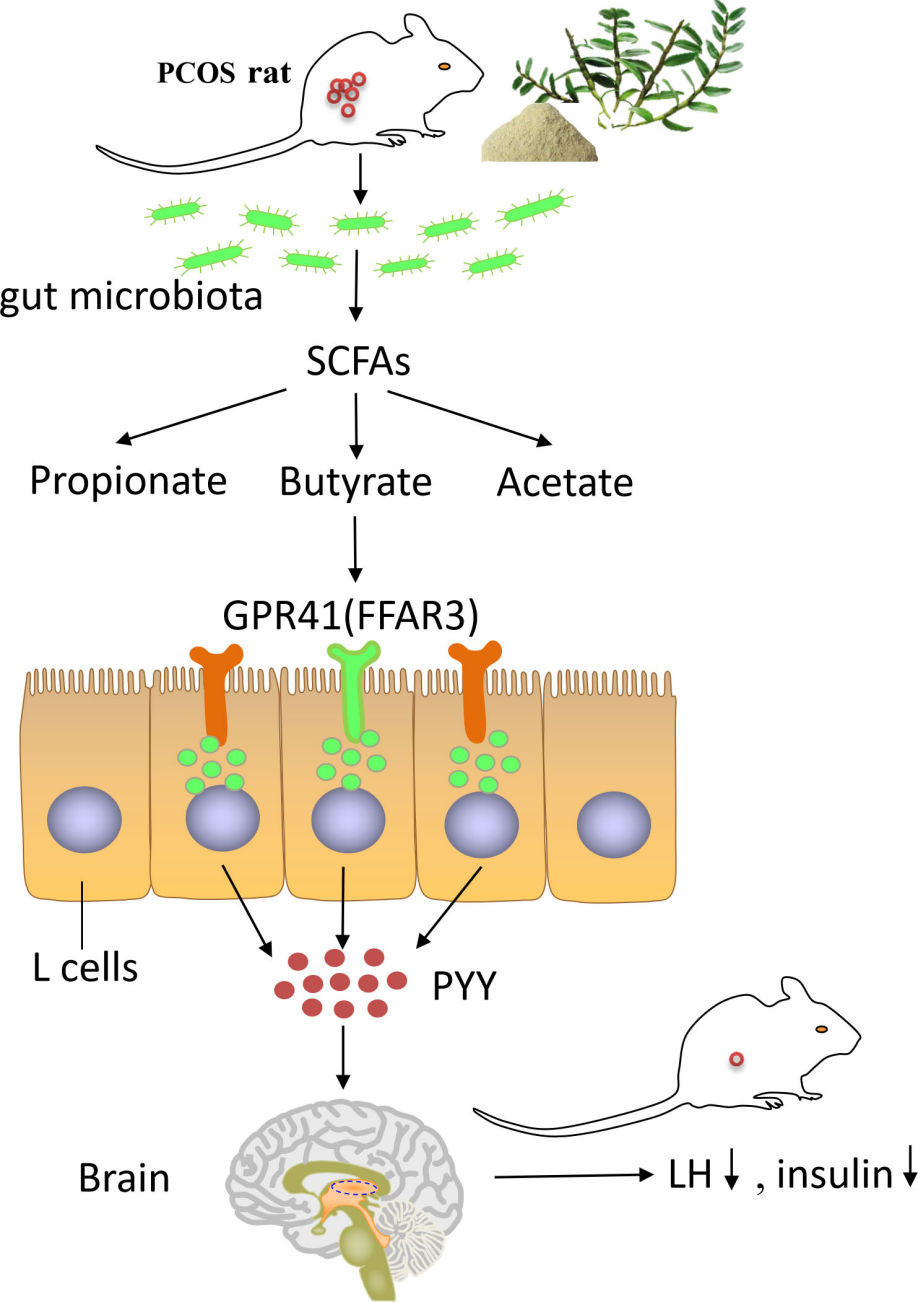
The summary of how DOP alleviates PCOS through gut–brain–ovary regulation. Dendrobium officinale polysaccharide can promote the remodeling of the gut microbiota and the production of butyrate bacterium in PCOS rats. High levels of butyrate through G protein-coupled receptor 41 to stimulate the secretion of PYY by entero-endocrine L-cells. PYY regulates the levels of steroid hormones through the gut–brain axis and ameliorates the polycystic ovary phenotype in PCOS rats.

The present study highlighted that DOP could reduce body weight, reverse endocrine disorder, restore the normal estrus cycle, improve polycystic ovary morphology, remodel the gut microbiota, and promote butyrate production in PCOS rats. The ameliorative mechanism is to improve the steroid hormone disorder, promote follicular development, and improve the symptoms of polycystic ovary through the butyrate-dependent gut–brain (GRP41-PYY) axis (**Figure 10**). These results provide theoretical references for DOP as a potential supplement for PCOS treatment.

## Data availability statement

The datasets presented in this study can be found in online repositories. The names of the repository/repositories and accession number(s) can be found below: NCBI PRJNA855132, available at https://www.ncbi.nlm.nih.gov/bioproject/PRJNA855132.

## Ethics statement

The animal study was reviewed and approved by Animal Welfare and Ethics Committee of Youjiang Medical University for Nationalities.

## Author contributions

Project administration, supervision, and writing—review and editing: QJ and YH; investigation, methodology, and writing—original draft: XF; methodology and data curation: DW; conceptualization: LH; software: HL; validation: BL. All authors contributed to the article and approved the submitted version.

## Funding

This work was supported by the Natural Science Foundation of Guangxi Province (Grant No. 2020JJB140033) in China.

## Conflict of interest

The authors declare that the research was conducted in the absence of any commercial or financial relationships that could be construed as a potential conflict of interest.

## Publisher’s note

All claims expressed in this article are solely those of the authors and do not necessarily represent those of their affiliated organizations, or those of the publisher, the editors and the reviewers. Any product that may be evaluated in this article, or claim that may be made by its manufacturer, is not guaranteed or endorsed by the publisher.

## Abbreviations

DOP, dendrobium officinale polysaccharide; PCOS, polycystic ovary syndrome; MET, metformin; CMC, carboxymethyl cellulose; NS, normal saline; LH, luteinizing hormone; FSH, follicle stimulating hormone; FBG, fasting blood glucose; T, testosterone; E_2_, estradiol; PYY, peptide tyrosine-tyrosine; GPR41, G protein-coupled receptor 41; SCFAs, short-chain fatty acids; CYP17A1, 17-a hydroxylase.
