# Supplementary material for: Dendrobium officinale polysaccharide ameliorates polycystic ovary syndrome via regulating butyrate dependent gut–brain–ovary axis mechanism
Source: Front Endocrinol (Lausanne). 2022 Aug 5;13:962775. doi: 10.3389/fendo.2022.962775 (PMC9389327; doi:10.3389/fendo.2022.962775)
Supplement: Supplementary file 1 [file DataSheet_1.zip › Supplementary 2/Supplementary 2/Presentation 1.PPTX]

## Slide 1
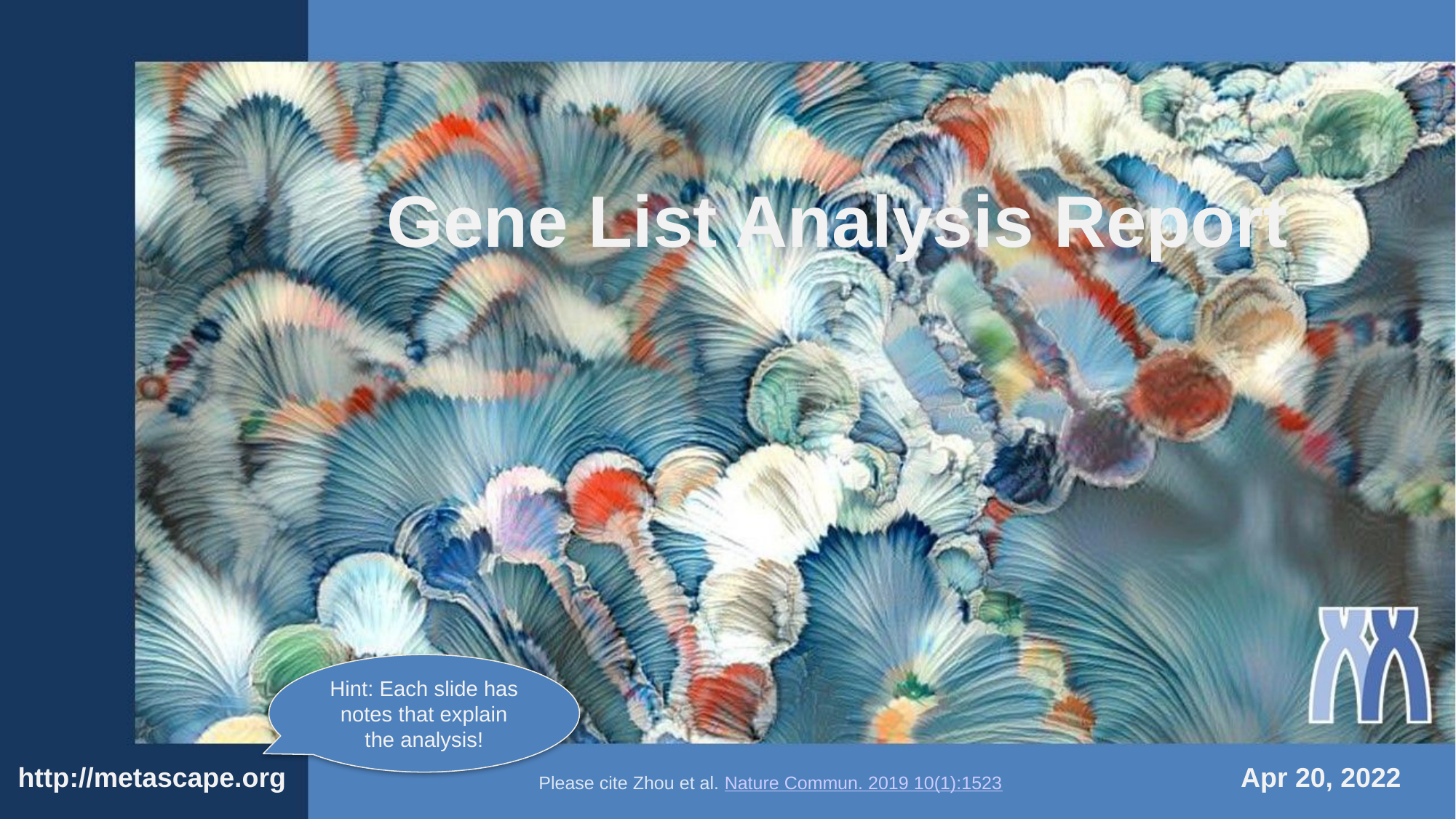

Gene List Analysis Report
Hint: Each slide has notes that explain the analysis!
http://metascape.org
Apr 20, 2022
Please cite Zhou et al. Nature Commun. 2019 10(1):1523

## Slide 2
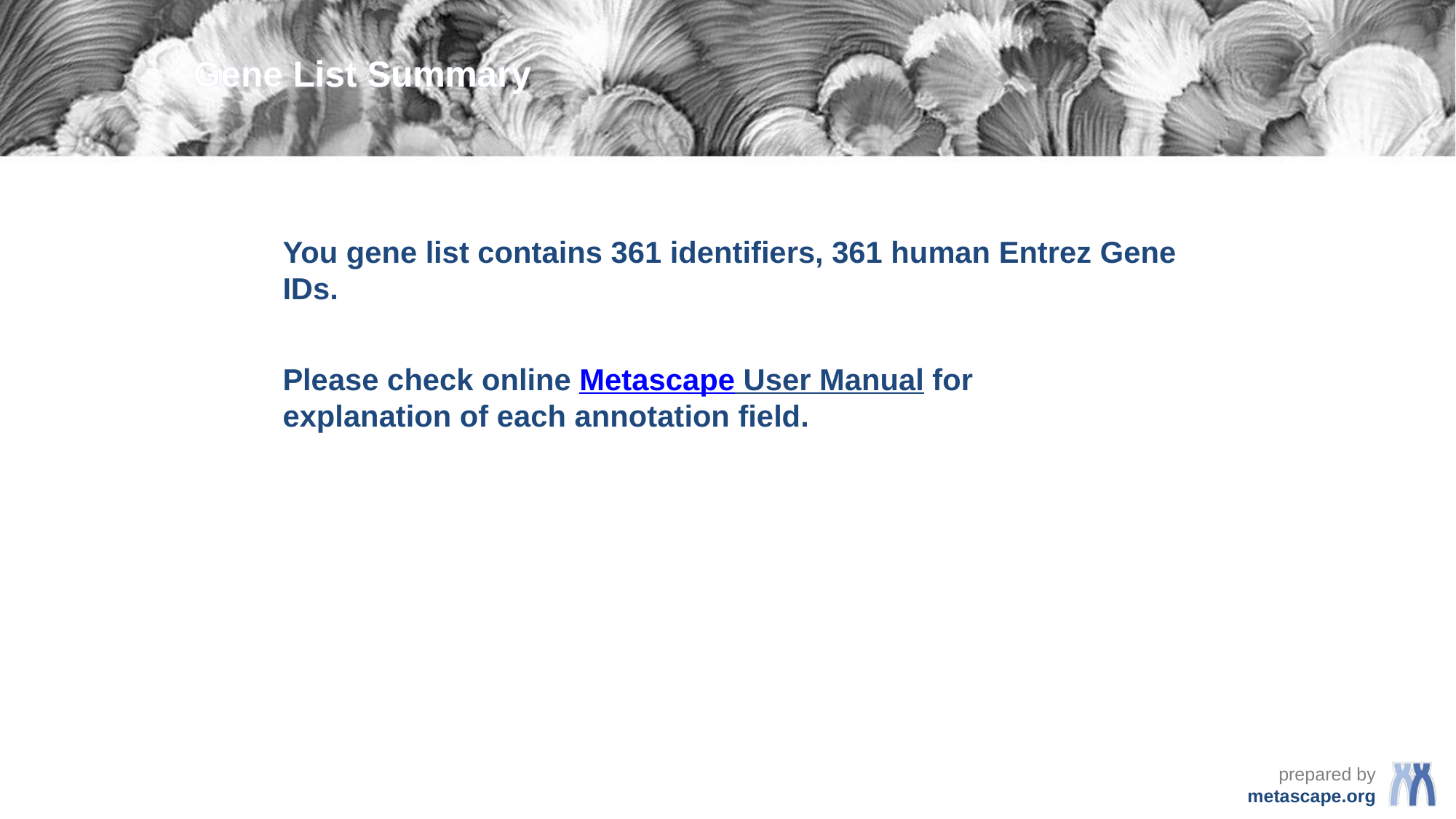

Gene List Summary
You gene list contains 361 identifiers, 361 human Entrez Gene IDs.
Please check online Metascape User Manual for explanation of each annotation field.

## Slide 3
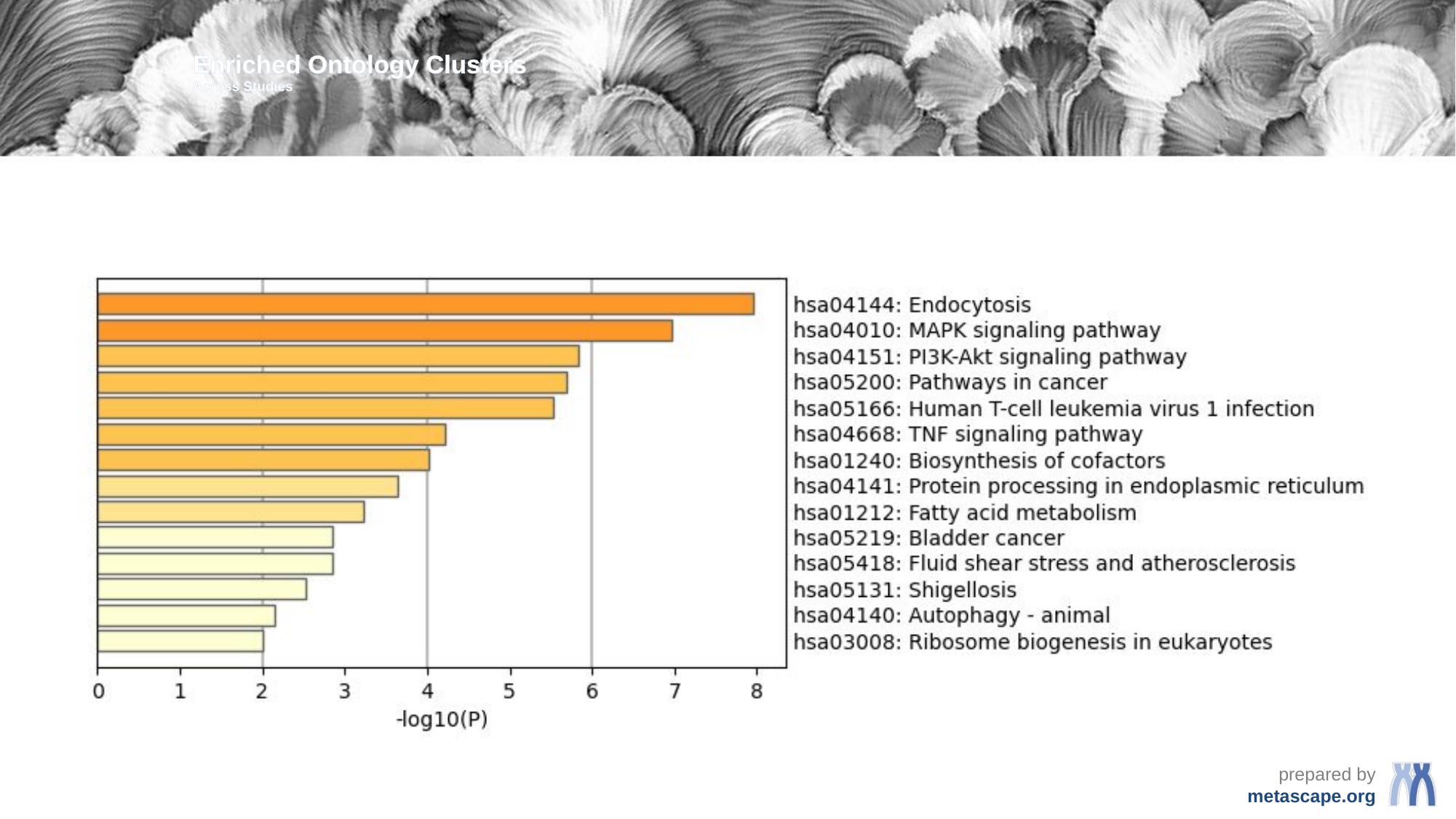

Enriched Ontology Clusters
Across Studies

## Slide 4
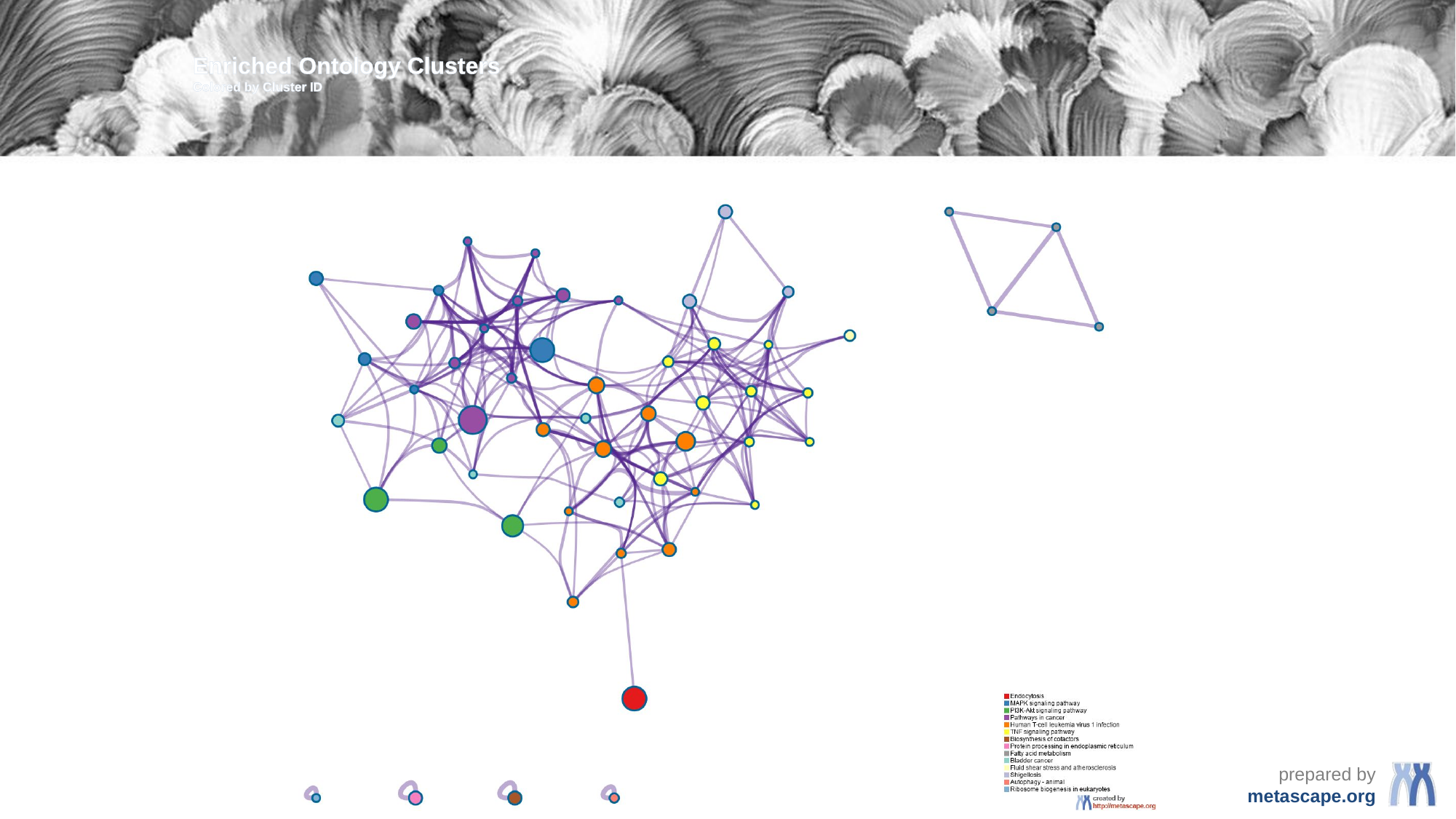

Enriched Ontology Clusters
Colored by Cluster ID

## Slide 5
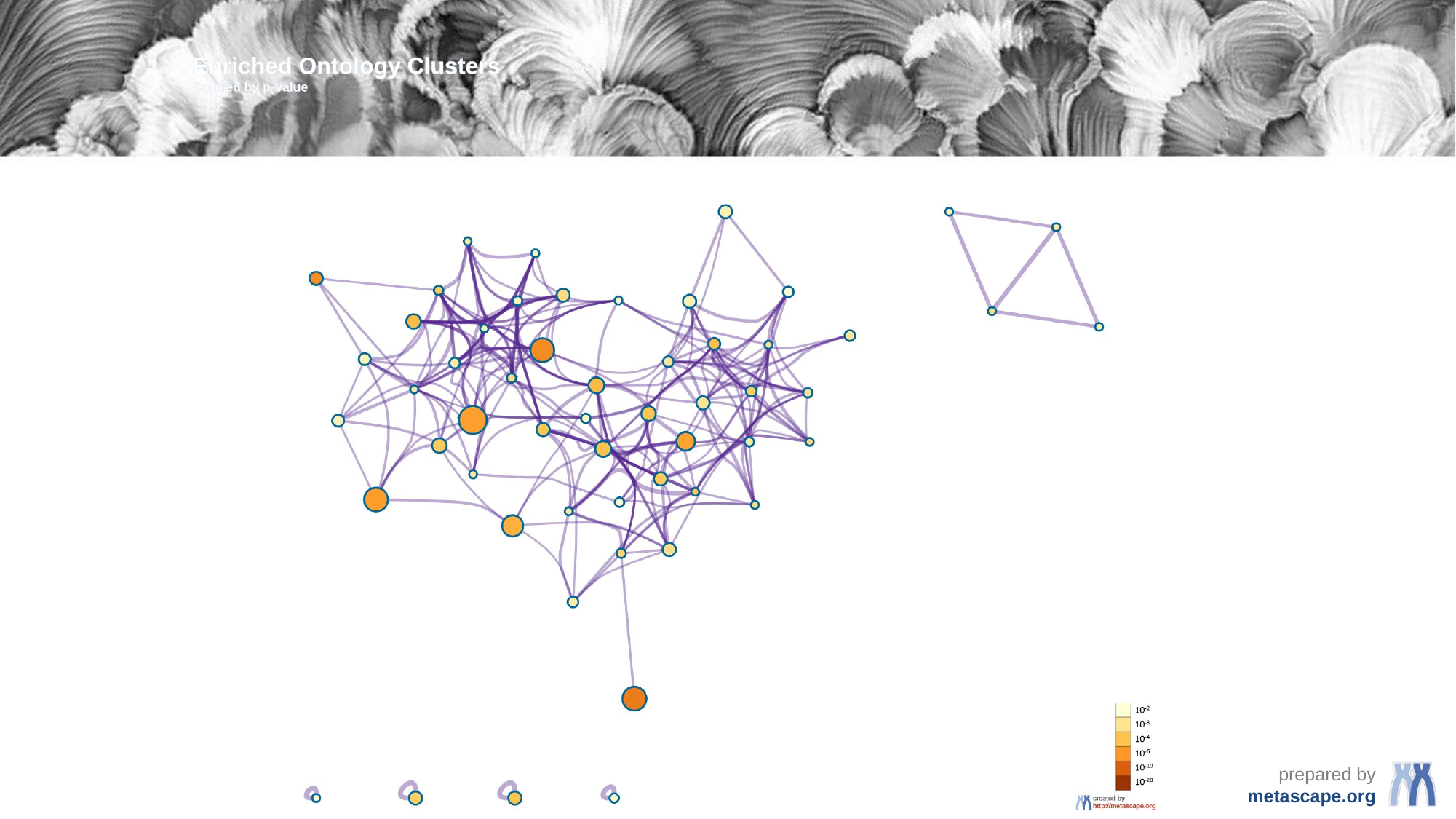

Enriched Ontology Clusters
Colored by p-Value

## Slide 6
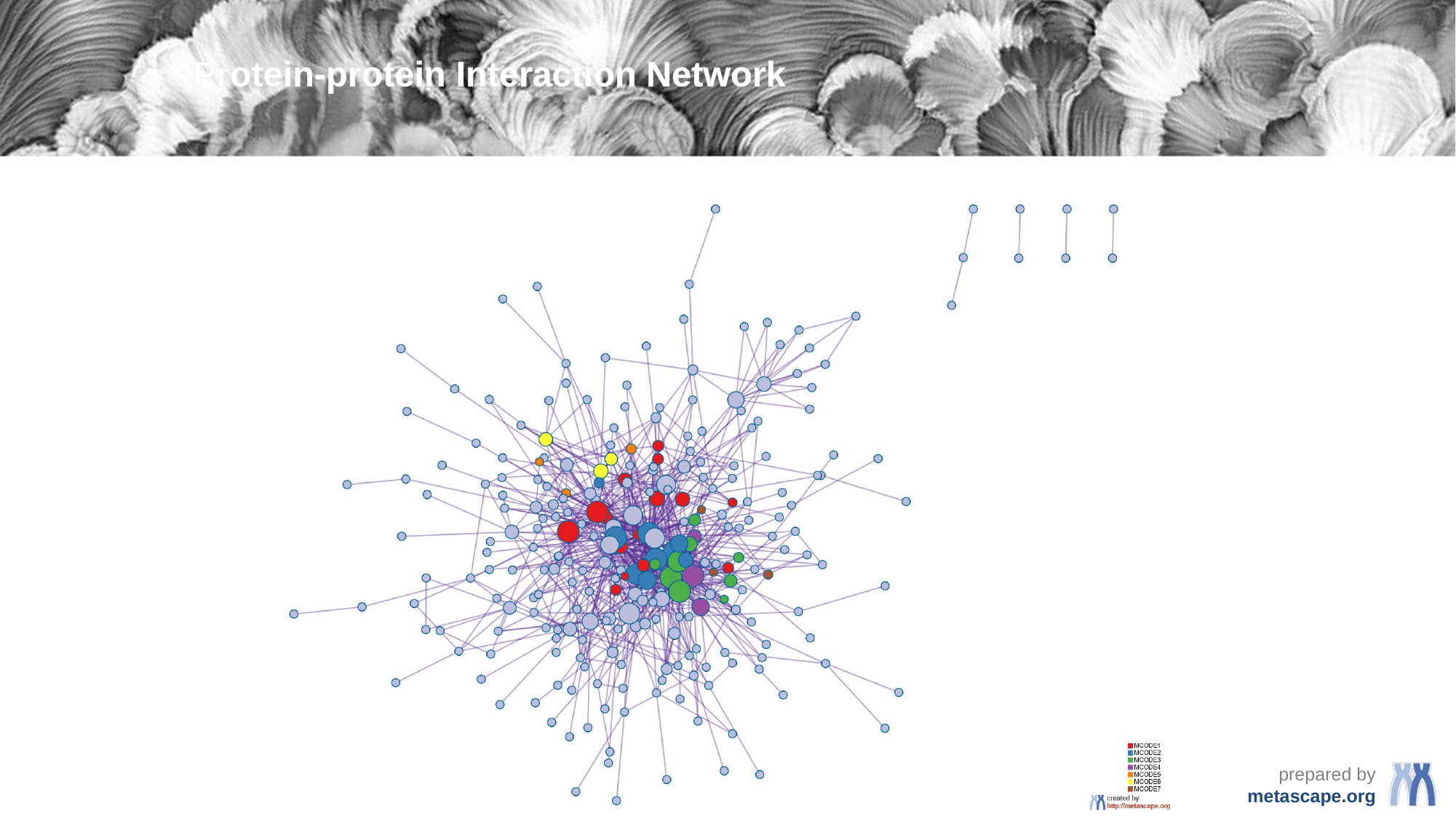

Protein-protein Interaction Network

## Slide 7
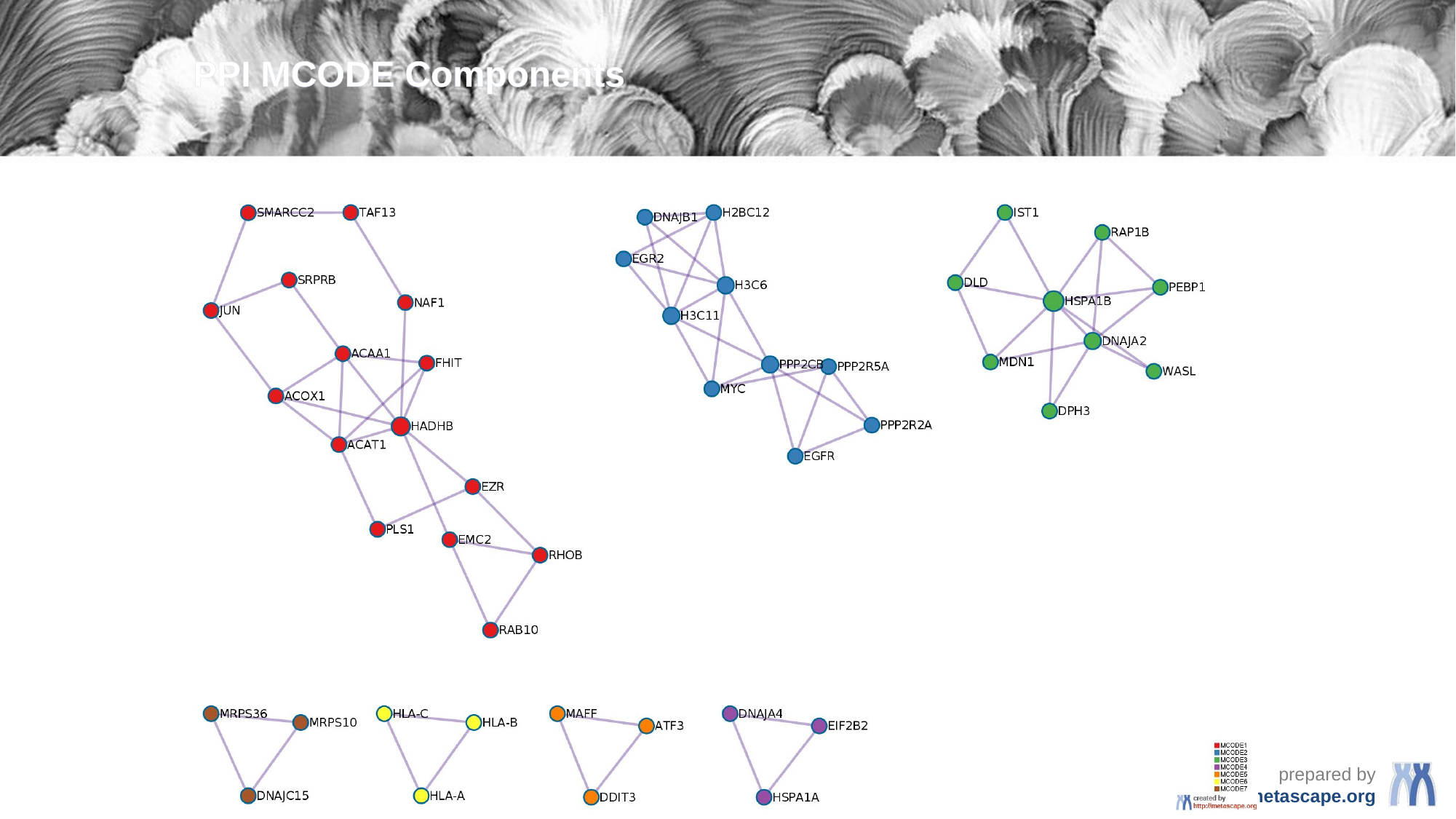

PPI MCODE Components

## Slide 8
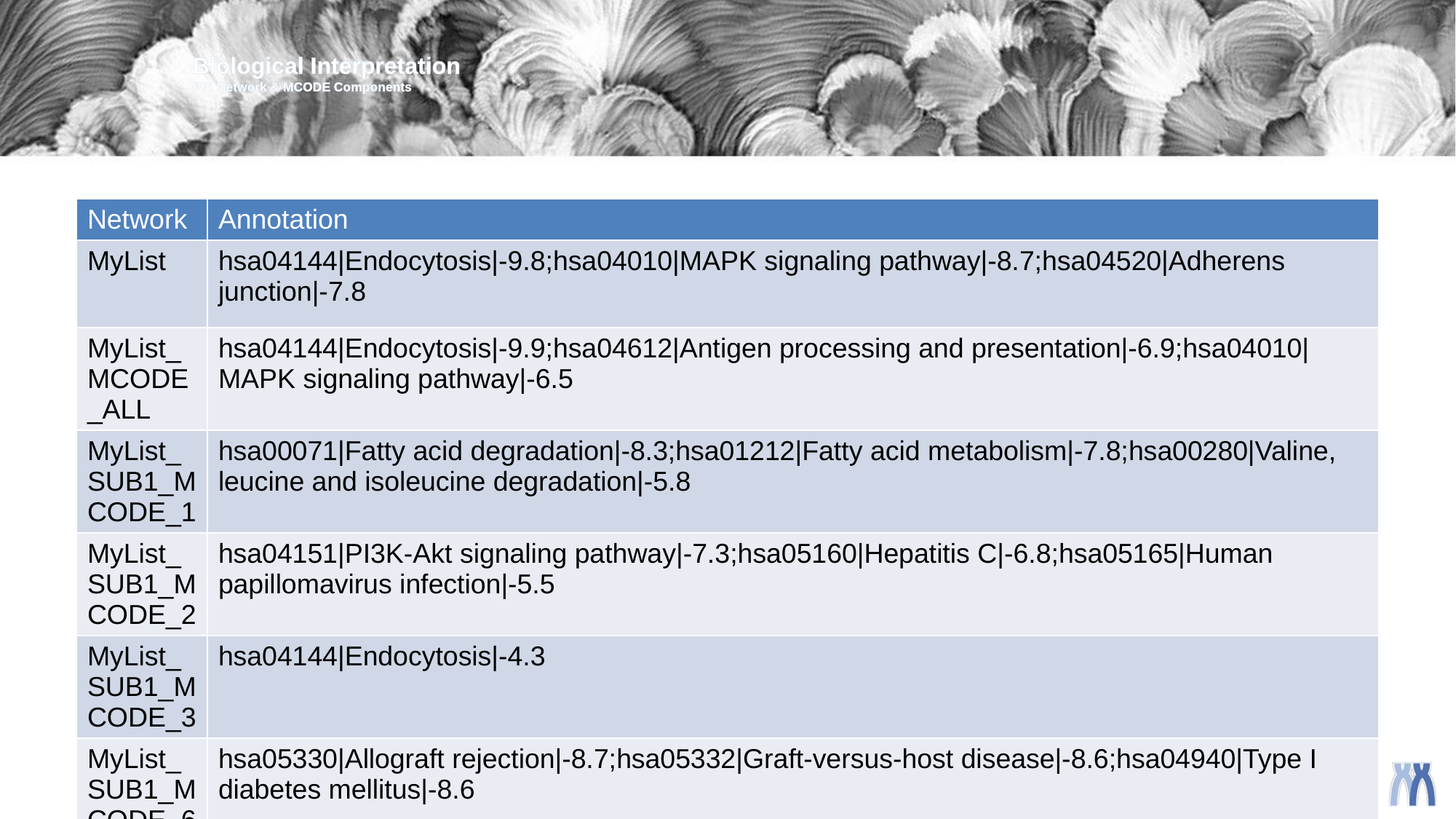

Biological Interpretation
PPI Network & MCODE Components
| Network | Annotation |
| --- | --- |
| MyList | hsa04144|Endocytosis|-9.8;hsa04010|MAPK signaling pathway|-8.7;hsa04520|Adherens junction|-7.8 |
| MyList\_MCODE\_ALL | hsa04144|Endocytosis|-9.9;hsa04612|Antigen processing and presentation|-6.9;hsa04010|MAPK signaling pathway|-6.5 |
| MyList\_SUB1\_MCODE\_1 | hsa00071|Fatty acid degradation|-8.3;hsa01212|Fatty acid metabolism|-7.8;hsa00280|Valine, leucine and isoleucine degradation|-5.8 |
| MyList\_SUB1\_MCODE\_2 | hsa04151|PI3K-Akt signaling pathway|-7.3;hsa05160|Hepatitis C|-6.8;hsa05165|Human papillomavirus infection|-5.5 |
| MyList\_SUB1\_MCODE\_3 | hsa04144|Endocytosis|-4.3 |
| MyList\_SUB1\_MCODE\_6 | hsa05330|Allograft rejection|-8.7;hsa05332|Graft-versus-host disease|-8.6;hsa04940|Type I diabetes mellitus|-8.6 |

## Slide 9
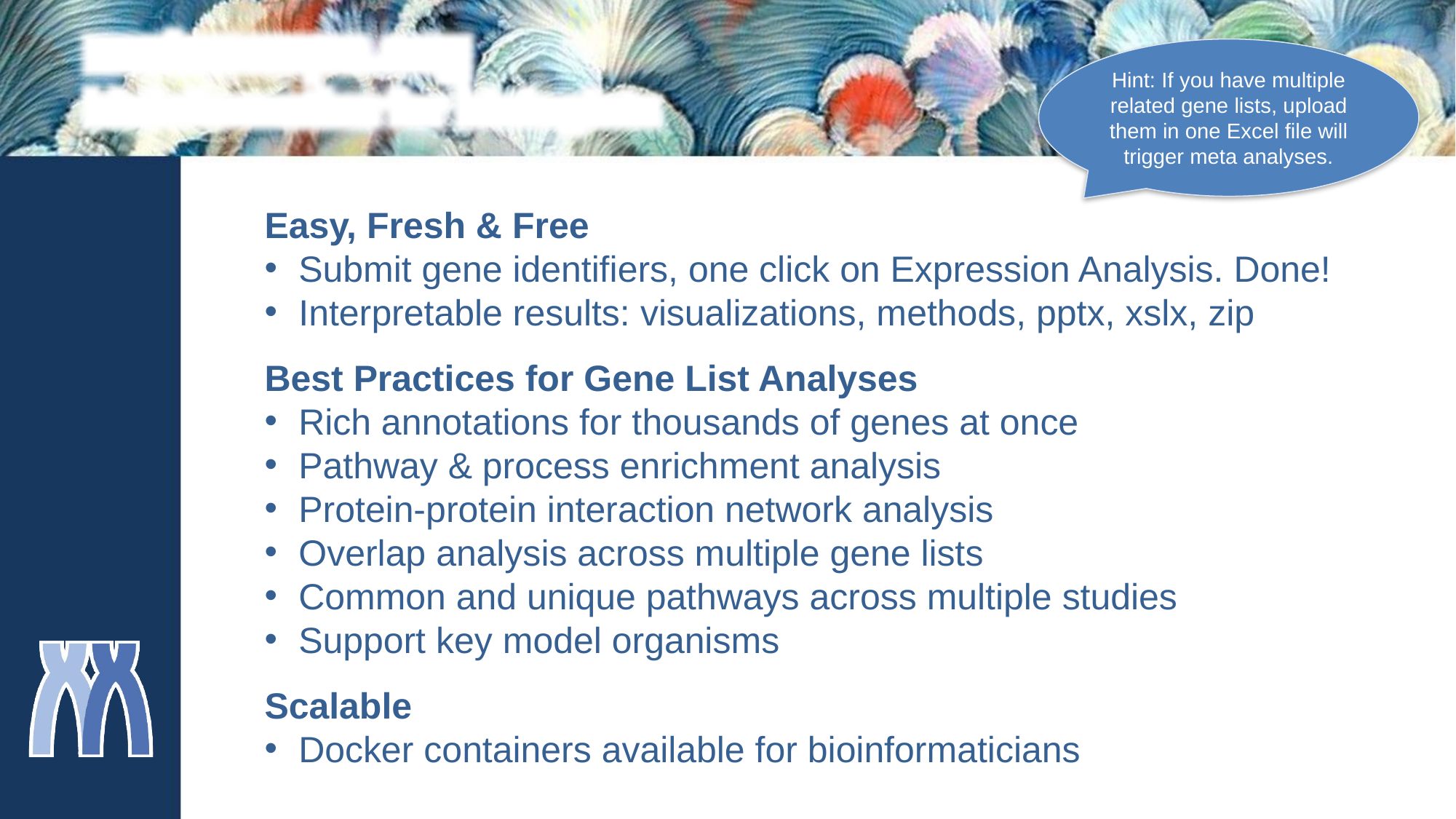

metascape.org
bioinformatics for biologists
Hint: If you have multiple related gene lists, upload them in one Excel file will trigger meta analyses.
Easy, Fresh & Free
Submit gene identifiers, one click on Expression Analysis. Done!
Interpretable results: visualizations, methods, pptx, xslx, zip
Best Practices for Gene List Analyses
Rich annotations for thousands of genes at once
Pathway & process enrichment analysis
Protein-protein interaction network analysis
Overlap analysis across multiple gene lists
Common and unique pathways across multiple studies
Support key model organisms
Scalable
Docker containers available for bioinformaticians
