# Supplementary material for: Dendrobium officinale polysaccharide ameliorates polycystic ovary syndrome via regulating butyrate dependent gut–brain–ovary axis mechanism
Source: Front Endocrinol (Lausanne). 2022 Aug 5;13:962775. doi: 10.3389/fendo.2022.962775 (PMC9389327; doi:10.3389/fendo.2022.962775)
Supplement: Supplementary file 1 [file DataSheet_1.zip › Supplementary 2/Supplementary 2/Table 1.DOCX]

TRIB1

HSPA1B

HSPA1A

INA

RAB21

CPPED1

AQP2

TDO2

ARMCX1

EGR2

GCNT3

LLPH

LLPH-AS1

LLPH

SSX2IP

TBC1D3I

TBC1D3E

LOC101060389

TBC1D3L

TBC1D3K

TBC1D3G

TBC1D3H

TBC1D3

TBC1D3P2

TBC1D3C

TBC1D3B

TBC1D3F

CHMP2B

DNAJA2

DNAJA2

SYAP1

CD24

SLIT2

ELMO1

NUS1

ICAM1

ZFAND6

P2RX5-TAX1BP3

P2RX5

TOR1AIP2

LAMA4

FGFR1

DPH3

IGBP1P1

SERPING1

EZR

ROS1

DDIT4

RAD51C

HES1

ITGB1BP1

LOC728323

FBXO25

SNAI2

C18orf21

PPBP

PPP1R10

AQP5

FRS3

SPRY2

FABP6

SRXN1

THBS2

CLYBL

NID1

MS4A6A

TBCA

GOPC

NEK6

NGRN

FAM3C

FAM35DP

FAM35BP

FAM35A

DENND6A

DMXL2

FSTL3

KDELC2

FAM107B

ABI3BP

GSTA5

C15orf38-AP3S2

AP3S2

EGR3

NR1D2

COQ10B

ELOVL4

ABR

STAG3L5P-PVRIG2P-PILRB

STAG3L2

STAG3L3

STAG3L1

STAG3

NT5E

SMIM15

C3orf70

TGFBR2

KLF4

PPP2CB

EIF2B2

CDV3

SSR3

TCTA

SERTAD1

ADGRD1

ASNS

C7orf25

IL24

LILRB2

DNAJC15

TMEM87A

NDEL1

CDON

AMZ2P1

DYNLT1

TAF13

SMUG1

RTN4RL1

KLRC2

POLR2H

COL6A1

POP5

CHMP5

CEMIP

NFATC4

DLD

MMP2

ZFP36

ATF3

PDGFRA

NEDD9

C3orf14

C6orf62

MRC1

SMARCC2

THAP2

GADD45G

LINC00467

PPP2R2A

GRAMD1A

BRINP3

UTP3

GSTA2

NDUFA1

USP45

DNAJB1

IST1

MIR21

F2RL2

CDK17

UVRAG

AMIGO3

MPRIP

TCEAL3

FAF2

ACOX1

SH3TC1

SRPRB

NFX1

GK3P

LAMTOR3

CELSR2

CEP295

LRRC75A-AS1

ISCA1

ATP13A3

ARFGAP3

SLCO2B1

EPM2A

MGC57346

SRR

ANAPC16

FAM103A1

GDAP1

ZNF701

TMEM116

IL18BP

ANGPTL1

WASL

VPS37B

MTHFD2

ZFAND2A

LDAH

FAM210A

REXO1

ITLN1

MRGPRX3

VEGFC

ZNF597

ZNF140

SLC25A33

EID1

SERPIND1

CREB3L2

RHOB

SMC6

PPP1R15A

PQLC1

SEPW1

GNPNAT1

NAT9

BEX2

MMP14

TOR1B

IL6

MED6

MAFF

TIMMDC1

DCAF11

GOSR1

STRAP

PLS1

MDN1

ZNF410

TANK

DNAJA4

ACAA1

TPMT

HIST1H3I

HIST1H3E

CYB5R4

SLC22A4

MTRF1L

FHIT

RAP1B

C1orf56

LAMTOR5

NAPG

NUMA1

FAM231A

LOC388692

PCTP

UBXN2A

EMC2

STAT6

DDIT3

IL1B

TOP1

OSER1

FAM216B

GADD45A

GLI3

FCGR2C

FCGR2B

FAM103A1

PTGS1

MGP

SLFN11

PEBP1

CAPRIN2

FJX1

DHX36

PSAT1

TMEM203

MPC1

ZNF793

SLC25A51

PLAC8

BIN1

PCDH9

STAG3L5P-PVRIG2P-PILRB

STAG3L2

STAG3L3

STAG3L1

PYURF

PIGY

BIN3

UXT

CAAP1

AFDN

MKL2

FBXL3

RBM12

CPNE1

HIST1H2BK

HSPA1B

HSPA1A

LYSMD3

RGN

SPRYD3

THEMIS2

PARGP1

GOLT1B

MAPRE3

ADAT1

VTN

PMM1

AMDHD1

LGALS9

CLIC5

ACAT1

IFI27

YES1

RARS

PIGX

EGFR

TMIGD3

ADORA3

TMIGD3

ADORA3

MYC

WLS

ENPP5

FBXO28

UBE2B

MRPS10

SBDS

RRAGC

LINC01089

TICRR

SVEP1

SVEP1

PRKDC

MRPS36

HLA-C

HLA-B

HLA-A

HLA-B

HLA-A

TCEAL4

POLR2K

GLRX2

STAB1

BPHL

RHPN1-AS1

SVIP

NADK

NAXD

YIPF5

RPF2

DAPK3

LZTR1

EMD

POP4

HADHB

BCL2L13

LYRM5

HHLA3

CRELD1

TSPAN5

COQ8B

1-Mar

NFS1

LPAR1

HTR2B

KLHL3

AIDA

SRPRA

CCDC92

SERPINI1

BLVRA

PPP2R5A

PCGF3

HMOX1

TMEM126A

ZNF263

TRADD

RNF141

RBP1

NFIX

GTF3C6

PEX2

AK3

MET

COPS2

NAF1

HEG1

GDF9

OLFML2A

PTPRS

SLC25A38

C20orf196

JUN

TMEM242

RAB10

CCDC159

OCR1

VPS26A

ZCCHC17
