# Supplementary material for: Dendrobium officinale polysaccharide ameliorates polycystic ovary syndrome via regulating butyrate dependent gut–brain–ovary axis mechanism
Source: Front Endocrinol (Lausanne). 2022 Aug 5;13:962775. doi: 10.3389/fendo.2022.962775 (PMC9389327; doi:10.3389/fendo.2022.962775)
Supplement: Supplementary file 1 [file DataSheet_1.zip › Supplementary 2/Supplementary 1/Supplementary 1.DOC]

Table1. The initial body weight(g) in rats.

| 0W | NS（g） | Model-1（g) | Model-2(g) | Model-3(g) |
| --- | --- | --- | --- | --- |
| 1 | **170** | **166.6** | **174.6** | **167.3** |
| 2 | **170.6** | **176.4** | **173.5** | **179.7** |
| 3 | **169.2** | **171.7** | **169.3** | **167.8** |
| 4 | **175.7** | **172.1** | **174.2** | **181.3** |
| 5 | **171.8** | **176.8** | **169.5** | **173.6** |
| 6 | **167.1** | **171.4** | **181.6** | **171.5** |
| mean | 170.7333333 | 172.5 | 173.7833333 | 173.5333333 |
| SD | 2.895283521 | 3.755529257 | 4.482595974 | 5.902767712 |

Note:Before the experiment starts, there is no significant difference in body weight between NS group and model groups (table1).
